# Floodgates

**DOI:** 10.1007/s11606-025-09563-x

**Published:** 2025-05-20

**Authors:** Sarah S. Fittro

**Affiliations:** Vanderbilt University Medical Center, Nashville, TN USA

“If her heart stops beating, does she want to be resuscitated?” the paramedic asked. I had been the one who called 911. The question made me feel like I had been punched in the throat. I burst into tears. The paramedics and firefighters stood in silence. It was as if months of emotions were released in one moment, as if being asked to state my mother’s code status was equivalent to being forced to face her impending death. Mom was still crying and mumbling incoherently as they rolled her out on a stretcher towards the ambulance. I paused answering their questions and watched her pass by. I felt like a failure, a failure as a caregiver. If only I had recognized her rising pain sooner, I could have avoided her pain crisis and this stressful ordeal of going to the emergency room.

We arrived to a full waiting room. Mom was left on the stretcher and parked in the hallway of the emergency department. The rooms were full and she would have to wait there. I was told I was not allowed to be with her since she did not have a private room. One of the paramedics escorted me to be with her despite hospital policy. I think he felt sorry for us.

As I approached her stretcher, it struck me how thin she had become over the last few months. Her oncologist offered trying one last treatment a few weeks earlier. We all agreed she would try the oral immunotherapy for a couple weeks and only continue if side effects were tolerable. The nausea was so horrendous she stopped taking it three days in.

And now, looking at my Mom waiting to be evaluated, I noticed her skin looked especially pale in the fluorescent lights, almost gray. She had a green emesis bag with some vomit already in it. She was given an antiemetic and fell into a deep sleep shortly thereafter; I am not sure if it was from the nausea medication, finally achieving relief from the pain, or a combination of both.

Eventually, Mom was moved into a private room, and the physician came in asking some basic questions. We told her how she had end-stage primary peritoneal cancer and her pain spiraled out of control over the course of the last 12 hours. I told myself all day that we simply got behind on her pain curve. Not once had I considered something else. The physician tried to warn me her peritoneal metastases could have caused a bowel obstruction or perforation. I either had not pieced together that possibility or subconsciously refused to believe something more ominous could have caused my Mom’s pain crisis. I assured the doctor she was just behind on her pain medications and she nodded sympathetically. I think she understood I needed to believe this was a fixable issue, at least in the short term.

Mom had the CT scan, which did not show perforation or obstruction. Her pain improved with intravenous medications, and I persistently asked how soon we could be discharged. With a mix of kindness and pity, the physician signed the discharge orders.

The following day, we enrolled her in hospice. Mom was in their care for just under four weeks. Her pain was managed well. She continued to lose weight. Her bones became more pronounced. I did not notice the day by day changes in her physical appearance, though when looking back at pictures, it was quite startling how emaciated she became. It was certainly difficult watching her fade away; however, I look back at that month on hospice with a lens of gratitude. My family and I were able to spend dedicated time with our mother, sister, daughter, aunt and friend. We knew the end was imminent, and that made the time so much more important. We soaked up the sun in her back yard. We watched the sunsets together. We laughed at old stories. We cried. We celebrated one last Mother’s Day with her. I got married a few months earlier than planned so she could be there.

Mom died on a Saturday. She had stopped eating or drinking a few days prior. Her breathing pattern had changed overnight and we knew it was coming. My sisters and I huddled around and held her. Her breaths became more sporadic and eventually stopped.

I started clinical rotations of my third year of medical school on Monday, two days later. This was my choice. My medical school certainly would have granted a longer leave for me, but I was determined to graduate on time and was worried extending my leave would jeopardize that. I had already taken three months of leave to be her caregiver. As I walked into the hospital Monday morning dressed in my hospital grade scrubs for my surgical clerkship, I felt a mix of sadness and empathy knowing I had a glimpse of what it was like to be a caregiver. To feel hopeless at times. To hold on to any sense of hope with an ironclad grip at other times.

Now a second-year resident, I often find myself reflecting on my time as a caregiver. I do not know what it is like to die of cancer, but I do know what it is like to watch a loved one die of cancer. When I ask a patient or their loved one about code status, I pause to consider what the impact of this question could have. For me, that moment was the one that opened the floodgates, the floodgates of years of emotions as a caregiver. How many other questions do we routinely ask in medicine that have the potential to trigger overwhelmingly emotional responses? The burden patients and their families carry are not something we can eliminate as physicians, but rather something we have an obligation to acknowledge and support.

Every patient and caregiver deals with a floodgate of emotions, and it is simply a matter of why and when this gate will open.

